# *Stephanosporamayana* (Stephanosporaceae, Russulales), a new sequestrate fungus from Yucatán Peninsula, Mexico

**DOI:** 10.3897/mycokeys.48.31007

**Published:** 2019-03-08

**Authors:** Javier Isaac de la Fuente, Gonzalo Guevara-Guerrero, Iván ros-Ortega, Romeo Sánchez-Zavalegui, Iván órdova-Lara, Jesús arcía-Jiménez

**Affiliations:** 1 Tecnológico Nacional de México. Instituto Tecnológico de Ciudad Victoria. Blvd. Emilio Portes Gil #1301Pte. CP87010, Ciudad Victoria, Tamaulipas, Mexico Instituto Tecnológico de Ciudad Victoria Ciudad Victoria Mexico; 2 Tecnológico Nacional de México. Instituto Tecnológico de la Zona Maya, Carretera Chetumal-Escácerga, km 21.5, CP 77965, AP 207, Ejido Juan Sarabia, Quintana Roo, Mexico Instituto Tecnológico de la Zona Maya Ejido Juan Sarabia Mexico; 3 Tecnológico Nacional de México, Instituto Tecnológico de Chetumal. Av. Insurgentes # 330, Col. David G. Gutiérrez, CP 77013, Chetumal, Quintana Roo, Mexico Instituto Tecnológico de Chetumal Chetumal Mexico; 4 Unidad de Biotecnología, Centro de Investigación Científica de Yucatán, CICY. Calle 43, Col. Chuburná de Hidalgo, CP 97025, Mérida, Yucatán, Mexico Unidad de Biotecnología Mérida Mexico

**Keywords:** Campeche, Macrofungi, Quintana Roo, tropical truffles, truffle-like fungi

## Abstract

*Stephanosporamayana* is presented as a new species from the Yucatán Peninsula, Mexico. This species is distinguished by the yellowish pileus, basidiospores with a small corona (4–6 × 1–2.5 µm), and variable size (8.0–17.0 × 6.0–11.0), thin pileus (21–40 µm) and the ecological association to lowland forest with *Haematoxylumcampechianum*, *Gymnopodiumfloribundum*, *Coccolobadiversifolia*, *Metopiumbrownei* and *Pinuscaribaea*. It differs from the American species of *Stephanospora*, like *S.michoacanensis* and *S.chilensis*, by its larger basidiospores. Descriptions, photographs and discussions are presented.

## Introduction

The species within *Stephanospora* Pat. were previously accommodated in Hymenogastraceae Vittad by Cunningham (1979) as *Octaviania* Vittad. and also in Octavianiaceae Locq. ex Pegler & T.W.K. Young by Pegler and Young (1979), due to the spiny basidiospores. They also included *Hydnangium* Wallr., *Sclerogaster* R. Hesse and *Wakefieldia* Corner & Hawker. Nevertheless, Overwinkler and Horak (1979) placed these species in the family Stephanosporaceae Overwinkler & Horak along with *Lindtneria* Pilát. due to the spines around the basidiospore base, forming what is called a corona. Actually, Stephanosporaceae includes both sequestrate and resupinate species with or without a corona (Martín et al. 2004; Vidal 2004; Castellano et al. 2007; Lebel et al. 2015). *Stephanospora* is a genus with sequestrate species characterized by the subhypogeous habit, spiny or crested basidiospore ornamentation, and the conspicuous corona at the basidiospore base. Most of the species have a yellowish to orange pileus, pale-orange, olive-grey to pale-brown hymenophore and lack a stipe (Castellano et al. 1986; Pegler et al. 1993; Montecchi and Sarasini 2000; Vidal 2004). According to Lebel et al. (2015), 15 species are recognized worldwide.

Most *Stephanospora* species grow in association with broadleaf trees in Oceania (Cunningham 1979; Bougher and Lebel 2001; Lebel et al. 2015) or temperate forest in Europe (Palacios and Lakisbar 1991; Pegler et al. 1993; Vidal 2004; Fraiture and Novello 2013) and America (Vidal 2004; Guevara-Guerrero et al. 2015). Some additional undescribed species and genetic sequences were mentioned by Lebel et al. (2015) from Belize, Costa Rica and the Caribbean. In the USA, no species have been described from fruiting bodies, but DNA sequences have been included in a couple of analyses (Edwards and Zak 2010; Lebel et al. 2015). Most species can be found growing under mycorrhizal trees species such as *Podocarpus*, *Eucalyptus*, *Quercus* or *Pinus*, but no evidence of ectomycorrhizal associations has been observed (Tedersoo et al. 2010). The genus is represented in Mexico so far by a single species, *S.michoacanensis* Guevara & Castellano from central Mexico (Guevara-Guerrero et al. 2015).

In recent mycological exploration conducted by us on the Yucatán Peninsula in southern Mexico, some interesting sequestrate fungi were found, collected and identified as *Stephanospora*. The specimens were collected under *Haematoxylumcampechianum* L., *Gymnopodiumfloribundum* Rolfe, *Metopiumbrownei* (Jacq.) Urband, and *Pinuscaribaea* Morelet in lowland forest and pine savanna. Due to the basidiospore size, small corona, the association to lowland forest and pine savanna, and a molecular analyses of DNA we conclude that it is a novel species and we propose it as *S.mayana* de la Fuente, García-Jiménez, Guevara-Guerrero & Oros-Ortega.

## Methods

### Sampling data

**Figure 1.**
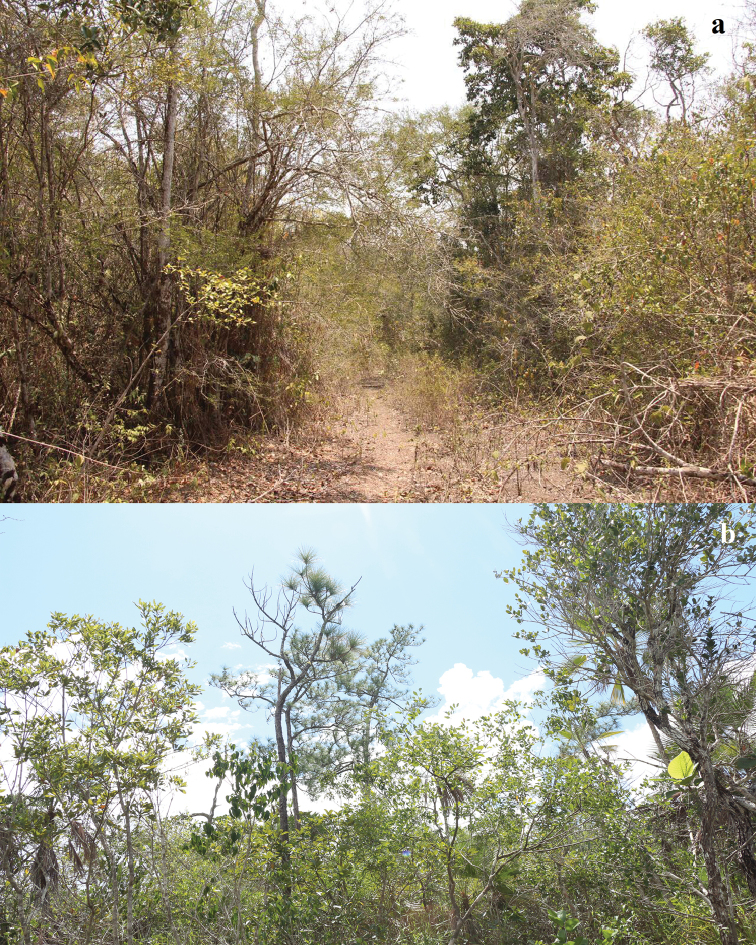
Habitat of *Stephanosporamayana*. **a** Lowland Forest at Blasillo **b** pine savanna at Xnohá.

Basidiomata were collected at Calakmul municipality in the state of Campeche and Othón P. Blanco municipality, in the state of Quintana Roo, Mexico. The vegetation is a disturbed lowland forest with *Coccolobadiversifolia* Jacq, *M.brownei*, *H.campechianum*, *G.floribundum*, and *Acoelorraphewrightii* (Griseb. & H. Wendl.) H. Wendl. ex Becc. (Valdéz and Islebe 2011) and pine savanna with *P.caribaea*, *C.diversifolia*, *Curatellaamericana* L., *Crescentiacujete* L., and *Byrsonimacrassifolia* (L.) Kunth (Macario and Sánchez 2011) (Fig. 1). Methods for collecting, sampling and describing sequestrate fungi were used (Castellano et al. 1986). Hand cuts sections were made from dried specimens mounted in KOH 5% and Meltzer reagent for microscopic description. Colour terminology was according to the Handbook of Colour (Kornerup and Wanscher 1978). All the specimens were curated and deposited at the mycological herbarium José Castillo Tovar of Instituto Tecnológico de Ciudad Victoria (ITCV).

### Molecular analysis

For DNA extraction from basidiomata tissue we used the protocol reported by Cordova et al. (2014). Briefly, 0.1 g of the tissue was pulverized in liquid nitrogen and 1 ml extraction buffer of CTAB (20 mM EDTA pH 8.0, 100 mM Tris-HCl pH 8.0, 2% CTAB, 1.4 M NaCl, and 2-mercaptoethano) was add and incubated for 20–30 min at 65 °C and then vigorously mixed with a solution of phenol-chloroform isoamyl alcohol. After centrifugation, the supernatant was precipitated using cold isopropanol and sodium acetate and then incubated at –20 °C for 1 h. The DNA was pelleted by centrifugation and dried at room temperature. Finally, the DNA was resuspended in 100 μL of nuclease-free ultrapure water. Quantity and quality of the DNA was estimated with a NanoDrop ND-1000 spectrophotometer (NanoDrop Technologies, Wilmington, DE, USA).

The ITS region of the ribosomal DNA was amplified using the primers ITS1F/ITS4B reported by Gardes and Bruns (1993). The final concentration of the PCR reaction was: 1× of MyTaq reaction buffer, 0.4 μM of primer, 40 ng of DNA and 1.5 Unit of MyTaq DNA polymerase (Bioline, USA Inc.). The PCR conditions used for amplification were according to Gardes and Bruns (1993). The PCR products were observed on a 1.5 % agarose gel stained with ethidium bromide and visualized by UV transillumination in a Gel-DOC (Bio-Rad) equipment. Bands amplified were removed and purified with the QIA quick gel extraction kit (QIAGEN). Purified PCR products were sequenced using automated equipment in Davis Inc., CA, USA. Both sides of the cloned inserts were sequenced. Sequences were aligned with MUSCLE (Edgar 2004). Alignments were manually checked and ambiguous regions were excluded. Sequences produced in this study are deposited in GenBank under accession number MK033630. A search of GenBank nucleotide databank (NCBI) for homologous sequences was performed by BLAST analyses.

Phylogenetic analyses was performed from sequences obtained from basidiomata. References sequences (Lebel et al. 2015) and consensus sequence were aligned using BioEdit version 7.0.4.1 (Hall 1999). The tree was built in MEGA X (Kumar et al. 2018) using maximum likelihood analyses and the Kimura 2-parameter model (Kimura 1980) of nucleotide substitution with bootstrap values based on 1000 runs. *Pilodermafallax* and *Atheliaarachnoidea* were used as outgroups (Lebel et al. 2015).

## Results

### Molecular analyses

**Figure 2.**
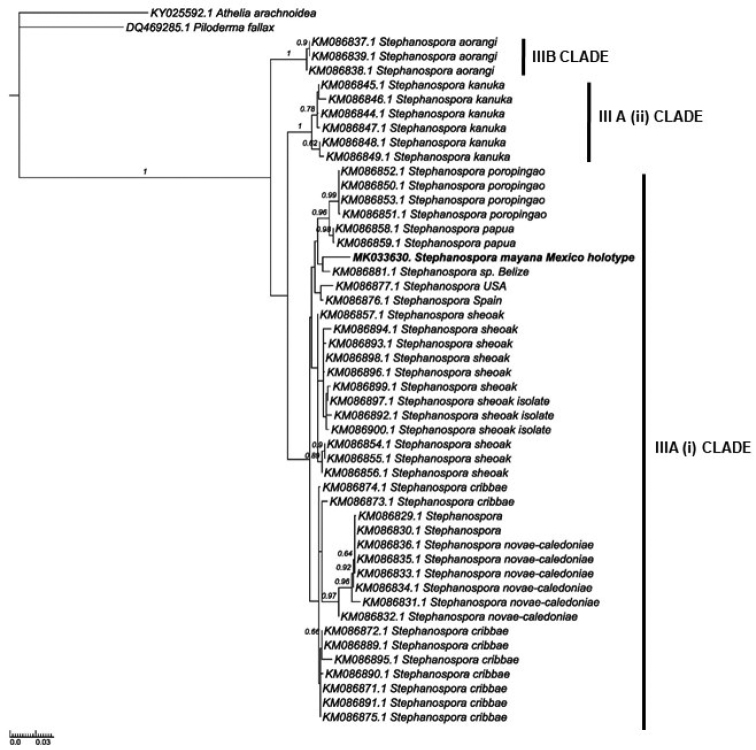
Phylogenetic tree inferred under the maximum-likelihood (ML) criterion from the ITS rDNA alignment corresponding the *Stephanospora* Clade III (i) dataset from Lebel et al. (2015). The tree was rooted using midpoint rooting. Numbers on the branches represent support values from 1,000 ML boostrap replicates. The branches are scaled in terms of the expected number of substitutions per site. Accession numbers in the sequence labels indicate sequences from GenBank.

A total of 48 sequences of *Stephanospora* species, including the new species, were analyzed (Fig. 2). The sequence consensus from the holotype clustered in the *Stephanospora* Clade III Subclade A (i) from Lebel et al. (2015). The designation of *S.mayana* as a new species is supported by ITS rDNA analyses and morphological features.

### Taxonomy

#### Stephanospora mayana

Taxon classificationFungiRussulalesStephanosporaceae

de la Fuente, García-Jiménez, Guevara-Guerrero & Oros-Ortega sp. nov.

828118

Figure 3a–f

##### Holotype.

Mexico: Campeche State, Calakmul Municipality, Blasillo town, 18°31'N, 88°18'W, 11 December 2017, de la Fuente (JF-397-ITCV), GenBank: MK033630.

##### Diagnosis.

*Stephanosporamayana* can be distinguished by the yellowish net-like pileus, the variable spore size (8.0–17.0 × 6.0–11.0 µm), thin pileus (21.0–40.0 µm) and the ecological association to lowland forest and pine savanna with *H.campechianum*, *G.floribundum*, *C.diversifolia*, *M.brownei*, and *P.caribaea*.

##### Etymology.

Named *mayana* in reference to the Mayan zone where this species was found.

##### Description.

Basidiomata hypogeous to subhypogeous, scattered, 3.0–15 × 2.0–6.0 mm, globose to subglobose, without rizomorphs or stipe. Pileus yellowish to slightly orange (5A6-30A3-6), bruising pale orange when touched, wet to dry, sometimes net-like, dehiscent, showing locules inside. Hymenophore brittle, grayish (5C4), with empty rounded to angular locules, reaching 0.5 mm long, sometimes with white short and slender hyphae projecting from pileus to locules, trama sometimes orange (5A7-5B7), odour and taste strongly fruity.

Pileus 21.0–40.0 µm thick, composed of loosely interwoven, slender to inflated hyphae, 1.7–4.2 µm in diameter, orange to pale orange-yellow in KOH, thin-walled. Hymenophoral trama irregular, 62.0–100.0 µm wide, composed of irregular, globose, isodiametric and compacted hyphae, 13.5–26.3 µm in diameter, hyaline to slightly yellowish in KOH, thin-walled. Basidia 24.2–30.5 × 9.5–11.1 µm, clavate to subclavate, hyaline in KOH, guttulate, 2-spored, with long sterigmata, reaching 7 µm long, thin-walled, collapsing after basidiospore development. Basidiospores (8.0–) 10.0–16.0 (–17.0) × (6.0–) 8.5–10.5 (–11.0) µm (*L* = 12.10, *W* = 9.31, *Q* = 1.30, *N* = 90) ellipsoid to subglobose, with truncate to acute spines projecting 2.0 µm long, forming ridges reaching 3.5 µm high, sometimes coalescing, with a complete to partial corona 4.0–6.0 × 1.0–2.5 µm long, sometimes with 2–4 projecting spines, 1.5 µm long, with hilar appendage conspicuous, reaching 3 µm long, bright yellowish in KOH, orange in Meltzer reagent, with greenish to yellowish cell wall, 1.5–2.0 µm thick. Hyphae from the locules hyaline, 3.0–5.0 µm diameter, thin-walled. Clamp-connections absent in all tissues.

**Figure 3.**
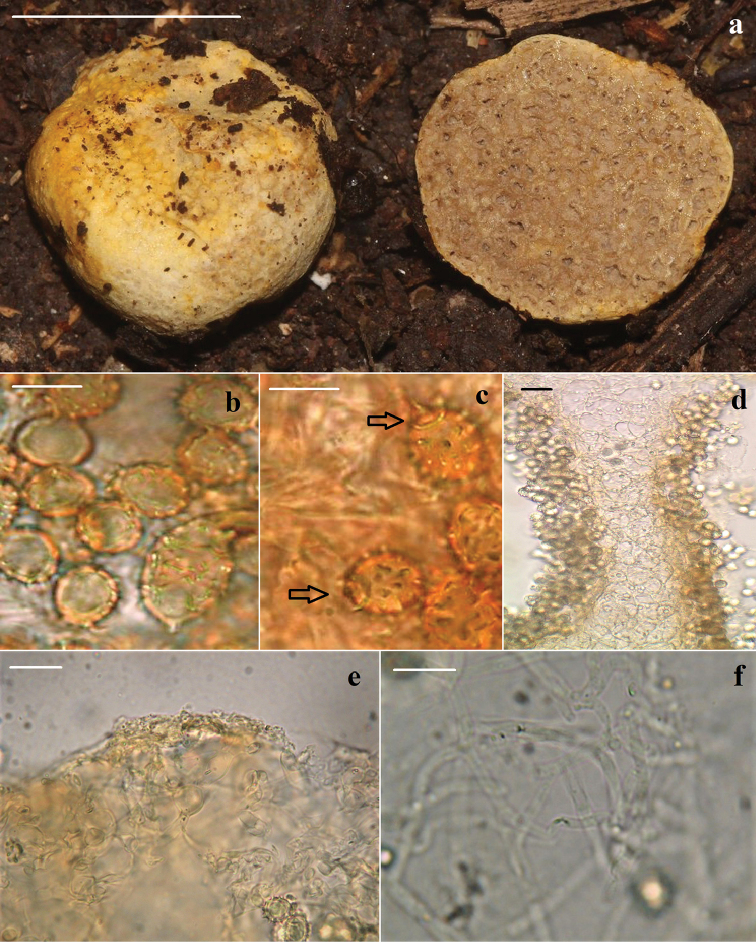
*Stephanosporamayana* (JF-397-ITCV-HOLOTYPE). **a** Basidiomata showing the pileus and hymenophore **b** basidiospores **c** corona **d** hymenophoral trama **e** pileus hyphae **f** hyphae from the locules. Scale bars: 10 mm (**a**); = 10 µm (**b, c, e, f**); 40 µm (**d**).

##### Distribution.

Known from the Mexican states of Campeche and Quintana Roo where it is associated to lowland forest and pine savanna under *G.floribundum*, *H.campechianum*, *M.brownei*, and *P.caribaea*.

##### Additional material examined.

Mexico, Quintana Roo, Othón Pompeyo Blanco municipality, Santa Elena Town, 18°30'N, 88°23'W, 07 October 2017, de la Fuente and Sánchez-Zavalegui 327 (Paratype); State of Campeche, Calakmul municipality, Xnohá town, 17°53'N, 89°10'W, 30 November 2017, de la Fuente 387 (paratype); Blasillo town, 18°31'N, 88°18'W, growing on abandoned termite mounds, 09 June 2018, de la Fuente 405 (paratype). (All in ITCV.)

## Discussion

This species belongs to the *Stephanospora* clade III A (i) following Lebel et al. (2015). All the species in this clade are characterized by having basidiospores with ornamentation that does not project more than 2.5 μm, basidia with sterigma up to 7 μm long, and a small corona that never surpasses 7 μm in width (Lebel et al. 2015). However, the Mexican material has larger basidiospores than any other species in this clade (up to 17 μm), unlike *S.poropingao* T. Lebel & Castellano, *S.papua* T. Lebel & Castellano, *S.novae-caledoniae* T. Lebel, Castellano & K. Hosaka, and *S.cribbae* T. Lebel & Castellano (up to 14 µm). *Stephanosporakanuka* T. Lebel & Castellano has similar pileus colour, sweet odour, and basidiospore length, but it has fine spines, an orange to yellowish hymenophore, and a fibrillose pileus (Lebel et al. 2015). *Stephanosporacribbae* T. Lebel & Castellano is similar to *S.mayana* in its yellowish pileus, corona size, and greyish hymenophore but differs in the smaller basidiospore size, the fibrillose pileus, and the coconut odour (Lebel et al. 2015). *Stephanosporamichoacanensis* differs from *S.mayana* in having smaller basidiospores, the fruit odour absent, a cream colour pileus, and its association with oak-pine forest (Guevara-Guerrero et al. 2015). *Stephanosporachilensis* (E. Horak) J.M. Vidal differs in having an orange pileus and hymenophore, as well as smaller basidiospores (Vidal 2004).

**Table 1.** Comparative morphology of *Stephanospora* species in the clade IIIA (i) according to Lebel et al. (2015).

Taxa	* S. poropingao *	* S. novae-caledoniae *	* S. cribbae *	* S. papua *	* S. mayana *
Basidiomata size	5–25 mm	5–18 mm	5–25 mm	5–22 mm	3–13 mm
Pileus surface	Fibrillose	Smooth	Fibrillose	Irregular	Net-like
Pileus colour	Bright yellow, orangish-yellow to orangish-brown	Pale yellow to bright orange	Yellow to orange-yellow	Pale orange-yellow	Pale yellow
Hymenophore colour	Greyish-olive, olive-brown to yellow	White to pale yellow	Greyish olive to olive brown-yellow	Pale yellow	Cream, greyish olive
Odour	Not recorded	Faintly sweet	Faintly cocconut	Not recorded	Fruity
Basidiospores size	11–14 × 11–13 µm	11–14 × 09–12 µm	11–13.5 × 9.5–12 µm	09–11 × 07–8.5 µm	08–17 × 06–11 µm
Spines	Robust	Cylindrical, flattened to acute	Fine	Cylindrical or ﬂattened	Truncated to acute
Corona size	05–09 × 01–03 µm	03–05 × 01–02 µm	03–05 × 01–02 µm	04–05 × 01–02 µm	04–06 × 01–2.5 µm
Pileus thickness	100–150 µm	40–145 µm	80–130 µm	30–140 µm	21–40 µm
Distribution	New Zealand, northwestern North Island	New Caledonia	Australia, Victoria, Queensland New South Wales	Papua New Guinea	Southern Yucatán Peninsula, Mexico
Habitat	*Agathis*-broadleaf, podocarp-broadleaf forest	Mixed forest with *Nothofagus* spp	*Eucalyptus* and *Acacia* Woodland	Mixed forest with *Eucalyptus*	Lowland forest and pine savanna

*Stephanosporamayana* is in an unsupported clade with undescribed species from Belize (KM086881) and close to another unsupported clade with undescribed taxa from the USA and Spain (Lebel et al. 2015). Further collections and descriptions of taxa are required for Belizean and US material to better place this new species.

## Supplementary Material

XML Treatment for Stephanospora mayana
